# Supplementary figures and images for: Complex Sequencing Rules of Birdsong Can be Explained by Simple Hidden Markov Processes
Source: PLoS One. 2011 Sep 7;6(9):e24516. doi: 10.1371/journal.pone.0024516 (PMC3168521; doi:10.1371/journal.pone.0024516)

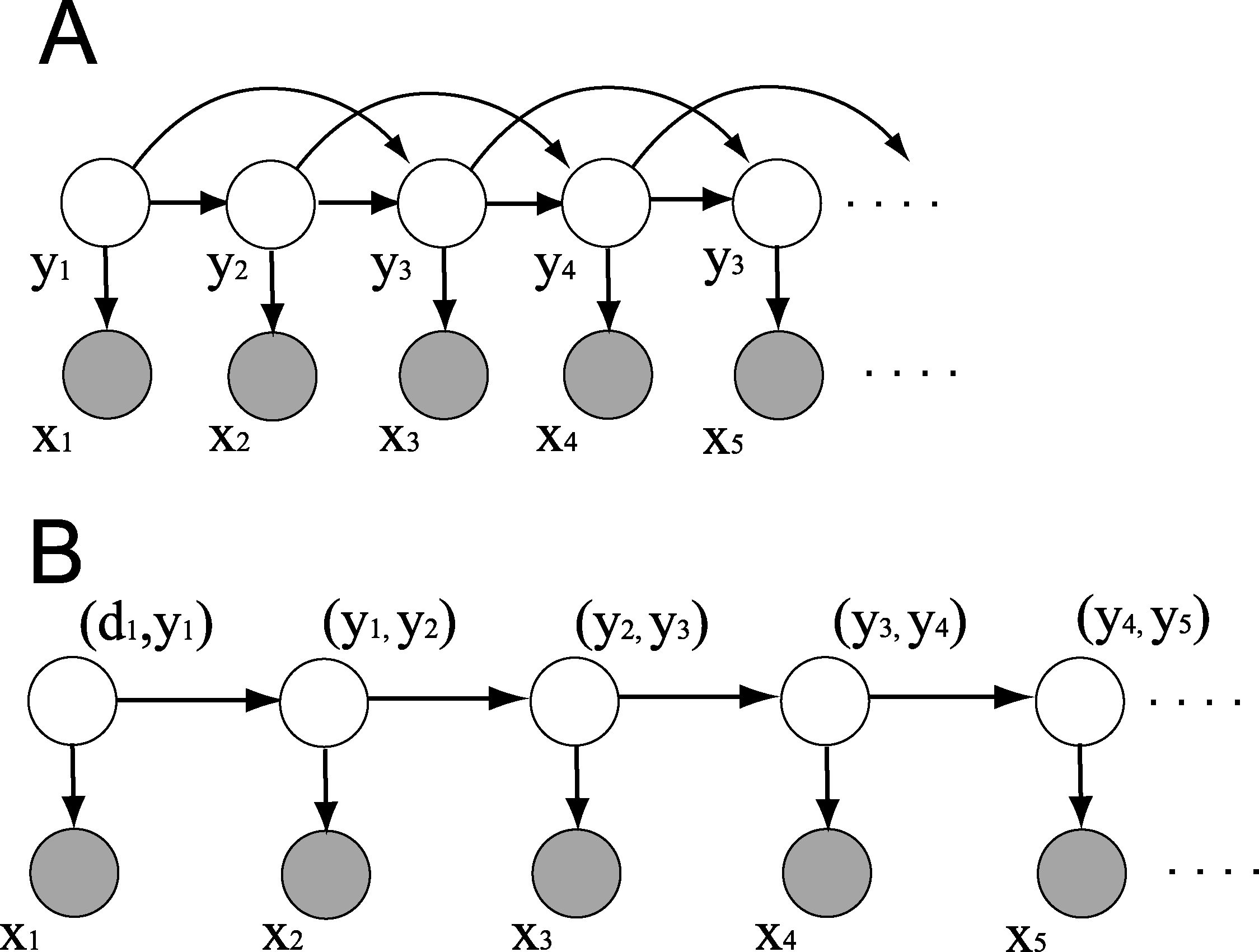

Supplement: Figure S1 — Graphical model representation for second-order HMM describing how parameter estimation for second-order HMM can be done. (A) Naive graphical model for second-order HMM. In this graph, we introduce a node (represented as a circle) for each random variable. For each conditional distribution, we add arrows to the graph from the nodes corresponding to the variables on which the distribution is conditioned. (B) Another representation of second-order HMM using context states that combine two states. (TIF) [file pone.0024516.s001.tif]
