# Supplementary material for: Complex Sequencing Rules of Birdsong Can be Explained by Simple Hidden Markov Processes
Source: PLoS One. 2011 Sep 7;6(9):e24516. doi: 10.1371/journal.pone.0024516 (PMC3168521; doi:10.1371/journal.pone.0024516)
Supplement: Table S1 — Statistics of songs recorded from individual birds. (DOC) [file pone.0024516.s002.doc]

| Bird ID | # syllable types | mean (maximum) bout length | # syllables with second order dependency, in non-repeated syllables  (in repeated syllables ) | # states which were found to be sufficient for first-order ^*1^ |
| --- | --- | --- | --- | --- |
| 1 | 9 | 52.3 (107) | 3 (1) | 12 |
| 2 | 7 | 94.6 (159) | 0 (2) | 10 |
| 3 | 9 | 89.5 (146) | 2 (4) | 15 |
| 4 | 9 | 80.2 (122) | 4 (0) | 15 |
| 5 | 8 | 123.9 (214) | 1 (2) | 10 |
| 6 | 6 | 99.7 (175) | 0 (2) | 11 |
| 7 | 6 | 93.3 (173) | 0 (2) | 9 |
| 8 | 10 | 87.3 (141) | 1 (2) | 11 |
| 9 | 11 | 86.3 (136) | 3 (1) | 13 |
| 10 | 9 | 88.0 (133) | 1 (2) | 13 |
| 11 | 9 | 113.7 (243) | 0 (2) | 14 |
| 12 | 8 | 108.8 (180) | 0 (4) | 16 |
| 13 | 14 | 83.3 (153) | 1 (3) | 14 |
| 14 | 14 | 48.0 (80) | 2 (1) | 15 |
| 15 | 12 | 115.8 (180) | 1 (2) | 14 |
| 16 | 15 | 64.6 (100) | 2 (3) | - ^*2^ |

**Table S1. Statistics of songs recorded from individual birds.**

*1: Defined as the number of states with which the first-order model gave a larger lower bound on log marginal likelihood than the largest one that was given by the second-order model.

*2: The best second-order model gave a lower bound which was slightly larger than the best first-order model.
